# Laser-induced photocatalytic degradation of tetracycline hydrochloride by ZnO/Cu_1.35_O composite material

**DOI:** 10.1016/j.isci.2025.114106

**Published:** 2025-11-17

**Authors:** Weiping Li, Xuemei Liang, Caixia Wu, Minglai Yang, Li Qin, Peng Jia, Helong Yu, Zhiqiang Cheng, Lijun Wang

**Affiliations:** 1 Intelligent Agriculture Research Institute, Jilin Agricultural University, Changchun, Jilin 130000, China; 2 College of Resources and Environment, Jilin Agricultural University, Changchun, Jilin 130000, China; 3 Changchun Institute of Optics, Fine Mechanics and Physics, Chinese Academy of Sciences, Changchun, Jilin 130000, China; 4 Jilin Provincial Science and Technology Innovation Center of Health Products and Medical Materials with Characteristic Resources, Changchun, Jilin 130000, China

**Keywords:** Physics, Materials science, Composite materials

## Abstract

Addressing antibiotic pollution in agricultural wastewater, we synthesized an S-scheme ZnO/Cu_1_._35_O heterojunction with over 52% Cu^+^ via ZIF-8 transformation, enhancing electron transfer and extending carrier lifetime to 18.3 ns. A low-energy laser achieved complete tetracycline mineralization, with a 5.1-fold rate increase by aligning wavelengths with the bandgap. Stability was sustained at 81.5% over cycles, with a Cu^+^/Cu^2+^ redox system. This introduces “laser wavelength-band engineering” for precise catalytic control, reducing energy consumption by 98% for efficient wastewater treatment.

## Highlights

• S-type ZnO/Cu_1_._35_O with 52% Cu^+^ and 2.6× carrier lifetime for superior charge separation
• Dual-laser (450/660 nm) excitation boosts the degradation rate constant by 5.1–6.3 times
• Cu^+^/Cu^2+^ redox buffer ensures stability (81.5% activity retention after 4 cycles)
• Achieves full TC mineralization with 98% lower energy consumption per unit pollutant

## Graphical abstract

**Figure:**
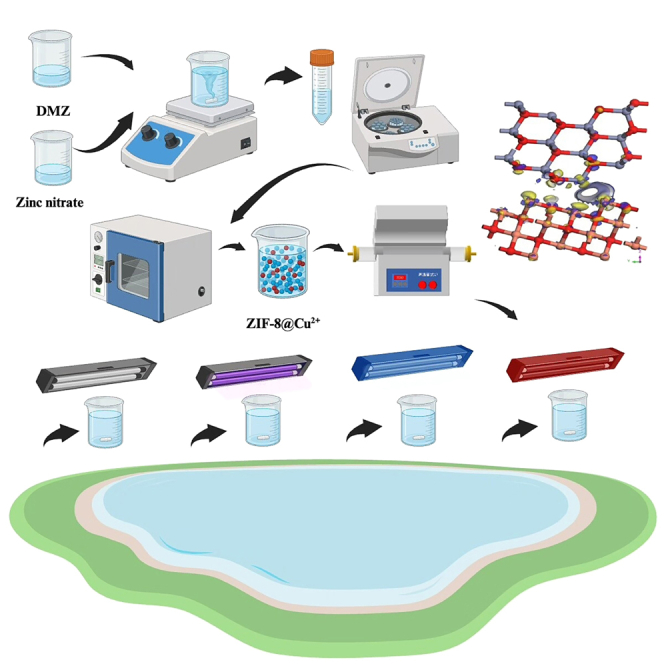

## Introduction

Agriculture is a vital pillar of the national economy, and its sustainable development is crucial for ecological security and human well-being. However, the excessive use of pesticides and fertilizers in agricultural production, along with improper disposal of livestock manure, has led to increasingly severe surface water pollution, posing a significant threat to ecosystems and public health.^1^^,^^2^^,^^3^^,^^4^ Traditional wastewater treatment technologies, such as adsorption, sedimentation, reverse osmosis, membrane separation, and biological treatment, are generally inefficient and prone to secondary pollution, failing to meet current sustainable development requirements for environmental protection and pollution control.^5^^,^^6^^,^^7^^,^^8^

Among various advanced oxidation processes (AOPs), semiconductor photocatalytic technology demonstrates significant potential. This technology can completely mineralize organic pollutants into CO_2_ and H_2_O under ambient conditions and pressure, with mild reaction conditions and generally no secondary pollution.^9^^,^^10^^,^^11^^,^^12^ Compared to technologies that rely on chemical reagent addition, such as ozonation and the Fenton process, photocatalysis uses light energy as the driving force, avoiding the residue of chemicals and subsequent treatment issues, making it more environmentally friendly and sustainable.^13^ An ideal high-efficiency photocatalyst should possess broad-spectrum absorption, efficient photogenerated charge separation capabilities, and a wealth of surface active sites.^14^^,^^15^

In recent years, the innovative strategy of using lasers as a photocatalytic light source has received widespread attention. Lasers possess characteristics such as high monochromaticity, strong coherence, and high energy density, which can precisely match the absorption bands of photocatalytic materials, significantly enhancing the efficiency of light utilization and quantum yield, thereby overcoming the inherent defects of traditional broad-spectrum light sources, such as energy dispersion and significant thermal loss.^16^^,^^17^^,^^18^^,^^19^^,^^20^^,^^21^^,^^22^^,^^23^^,^^24^^,^^25^^,^^26^^,^^27^^,^^28^^,^^29^^,^^30^^,^^31^

Metal-organic frameworks (MOFs), particularly zeolitic imidazolate frameworks (ZIFs), have garnered extensive research attention in the field of pollutant removal due to their high surface area, tunable pore structures, and excellent chemical stability.^32^^,^^33^^,^^34^^,^^35^^,^^36^^,^^37^^,^^38^^,^^39^^,^^40^ Notably, ZIF-8, as a representative example, demonstrates promising potential in the adsorption and photocatalytic degradation of dyes and organic pollutants.^38^^,^^39^^,^^40^^,^^41^^,^^42^^,^^43^ However, the poor conductivity and mechanical strength of ZIF-8 itself limit its practical applications.^42^^,^^43^^,^^44^^,^^45^^,^^46^ Constructing ZIF-8-based composite materials, such as those combined with metal oxides or carbon materials, can effectively improve their conductivity and stability, and significantly enhance photocatalytic activity.^47^^,^^48^^,^^49^^,^^50^ Furthermore, nanofiber materials with high porosity have been proven to have broad application prospects in the efficient treatment of colored wastewater.^51^^,^^52^^,^^53^^,^^54^

The aim of this study is to develop a laser-induced photocatalytic system based on ZIF-8 composite materials for the efficient degradation of antibiotic pollutants in agricultural wastewater. This strategy is dedicated to providing avenues for achieving greener outcomes and enhancing sustainability. low-energy pollution control. Additionally, this article systematically summarizes the key factors affecting photocatalytic degradation behavior and discusses effective strategies for enhancing photocatalytic performance, aiming to provide theoretical foundations and technical guidance for the design and practical application of innovative photocatalytic systems.

## Results and discussion

### Material structure and morphology characterization

**Figure 1:**
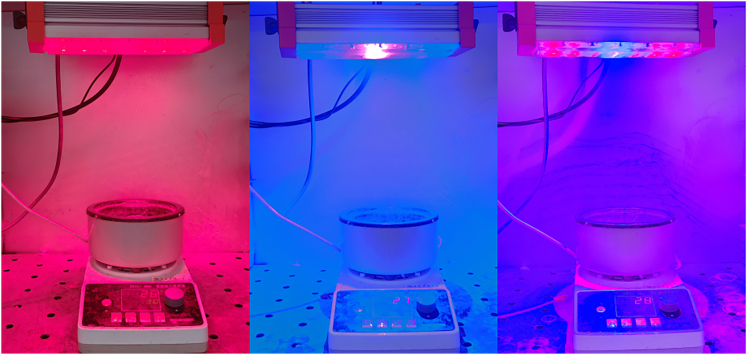
Degradation process of tetracycline hydrochloride under laser catalysis

**Figure 2:**
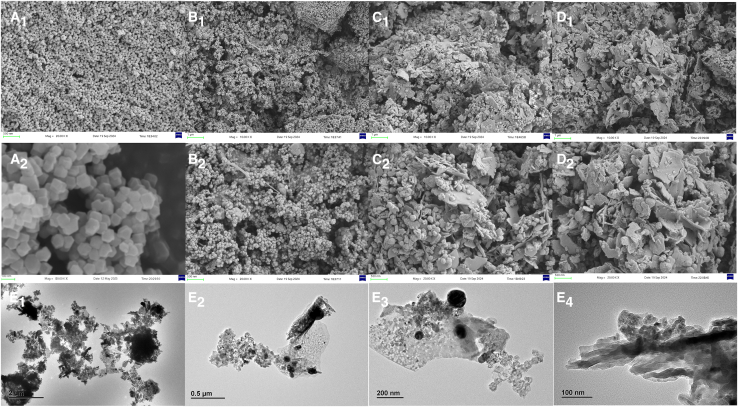
Scanning electron microscopy (SEM) and transmission electron microscopy (TEM) images of various materials (A) is the scanning electron microscopy (SEM) image of ZIF-8, (B) is the SEM image of ZnO, (C) is the SEM image of ZIF-8/Cu, (D) is the SEM image of ZnO/Cu_1_._35_O, and (E) is the transmission electron microscopy (TEM) image of ZnO/Cu_1_._35_O.

This experiment uses a 15W laser lamp developed by Zhejiang Changxin Optoelectronics Company as the light source, as shown in Figure 1. The central wavelength of the red laser is approximately 660 nm, and the central wavelength of the blue laser is approximately 450 nm. The morphology of the materials was characterized using scanning electron microscopy (SEM) and transmission electron microscopy (TEM) (Figure 2). Figure 2A shows that ZIF-8 synthesized with methanol as the solvent exhibits a dodecahedral structure with a smooth surface and sharp edges, which is consistent with previous research results. After calcination, the ZIF-8 structure decomposes, and a layered structure is formed after the incorporation of copper ions (Figure 2C). As the calcination progresses, the layered structure becomes more prominent, and a porous structure is formed between the layers (Figure 2D). The TEM results (Figure 2E) verify the layered porous morphology of ZnO/Cu_1_._35_O. This morphology provides a high specific surface area, facilitating the transport and separation of photogenerated carriers, thereby creating ideal conditions for the photocatalytic degradation reaction. This structural design significantly enhances the photocatalytic activity of the material and lays a material foundation for the efficient removal of organic pollutants.

X-ray photoelectron spectroscopy (XPS) analysis has elucidated the elemental composition and chemical states of ZIF-8, ZnO, ZIF-8/Cu, and ZnO/Cu_1_._35_O, providing pivotal evidence for understanding the formation mechanism of ZnO/Cu_1_._35_O. The analysis revealed that all samples exhibited a carbon contamination peak at 284.8 eV, with ZIF-8 and its derivatives displaying characteristic peaks for C–N/C–O and C=O bonds at 286.0 eV and 288.6 eV, respectively. In contrast, the carbon signal was significantly reduced in pure ZnO and ZnO/Cu_1_._35_O, indicating the decomposition of the ZIF-8 framework. Samples 1, 3, and 4 exhibited pyridinic nitrogen peaks at 398.5 eV, with the nitrogen signal in sample 4 suggesting that nitrogen-containing species were partially retained or transformed during the calcination process. A characteristic peak at 531.5 eV, attributed to surface adsorbed oxygen or oxygen vacancies, was observed in all samples, with an intensified peak in sample 4, indicating that the introduction of copper increased the concentration of oxygen vacancies. Copper signals were detected in samples 3 and 4, with a near 1:1 ratio of Cu^+^ to Cu^2+^, suggesting the presence of copper in the form of Cu_1_._35_O, which is characterized by abundant oxygen vacancies and mixed valence states. All samples demonstrated that zinc predominantly exists in the form of Zn^2+^, with samples 2 and 4 exhibiting similar spectral profiles, thereby confirming the presence of the ZnO matrix. The ZnO/Cu_1_._35_O composite material was synthesized through the calcination of ZIF-8/Cu precursors, during which the organic framework of ZIF-8 decomposed, releasing nitrogen-containing species, while zinc and copper species were oxidized. Copper exists in a mixed-valence state of Cu^+^ and Cu^2+^, closely integrated with the ZnO matrix. XPS results confirm that the successful synthesis of ZnO/Cu_1_._35_O is attributed to the thermally induced transformation of the precursors, with the material’s surface featuring an abundance of oxygen vacancies and a stable Cu^+^/Cu^2+^ valence state combination. These characteristics endow the material with potential applications in catalysis and sensing, particularly the synergistic effect between mixed-valence copper and oxygen vacancies, which can significantly enhance surface reactivity.

**Figure 3:**
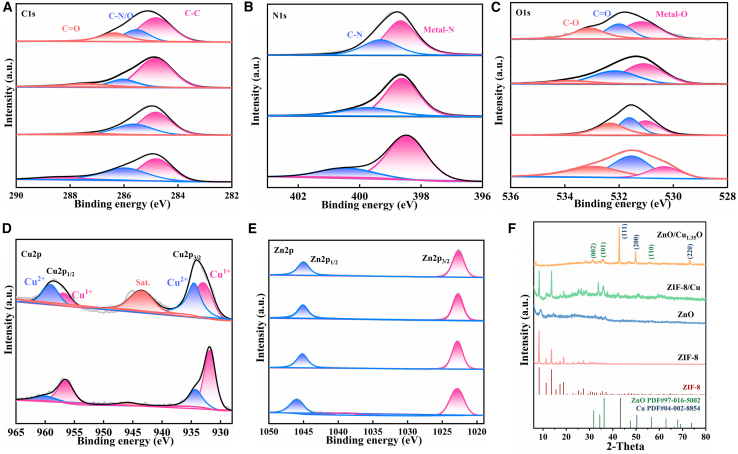
X-ray photoelectron spectroscopy (XPS) and X-ray diffraction (XRD) patterns of the four materials (A) presents the XPS spectra of carbon C for the four materials: ZIF-8, ZnO, ZIF-8/Cu, and ZnO/Cu_1_._35_O; (B) shows the XPS spectra of nitrogen (N) for the three materials: ZIF-8, ZIF-8/Cu, and ZnO/Cu_1_._35_O; (C) illustrates the XPS spectra of oxygen (O) for the four materials: ZIF-8, ZnO, ZIF-8/Cu, and ZnO/Cu_1_._35_O; (D) depicts the XPS spectra of copper (Cu) for the two materials: ZIF-8/Cu and ZnO/Cu_1_._35_O; (E) displays the XPS spectra of zinc (Zn) for the four materials: ZIF-8, ZnO, ZIF-8/Cu, and ZnO/Cu_1_._35_O; (F) presents the XRD patterns for the four materials: ZIF-8, ZnO, ZIF-8/Cu, and ZnO/Cu_1_._35_O.

X-ray diffraction (XRD) was employed to investigate the crystal structures of ZIF-8, ZnO, ZIF-8/Cu, and ZnO/Cu_1_._35_O, with results depicted in Figure 3F.The characteristic diffraction peaks of ZIF-8 were observed at 2θ values of 7.33°, 10.37°, and 12.78°, consistent with literature reports.^27^ After calcination, ZIF-8 partially transformed into ZnO, with XRD patterns exhibiting coexistence of diffraction peaks from both ZIF-8 and ZnO, indicating incomplete transformation. In the ZIF-8/Cu composite, copper ions were successfully doped into the ZIF-8 framework, causing changes in the intensity of diffraction peaks and the emergence of characteristic peaks for copper nanoparticles at 2θ values of 33.15°, 35.43°, and 41.87°. Further calcination of ZIF-8/Cu yielded the ZnO/Cu_1_._35_O composite, with peaks appearing at 43.32°, 50.35°, and 73.91° in its XRD spectrum corresponding to the (111), (200), and (220) crystal planes of Cu_1_._35_O, confirming the presence of this phase. In conjunction with XPS analysis (Figures 3A–3C), various carbon chemical bonds, including C=C, C–N/O, C–C, C–N, and Metal–O, were detected in the samples, indicating that carbonaceous materials generated from the decomposition of ZIF-8’s organic ligands during calcination are retained. These carbon species may serve as catalytically active sites or facilitate electron transfer, thereby positively impacting the catalytic performance of the material.

**Figure 4:**
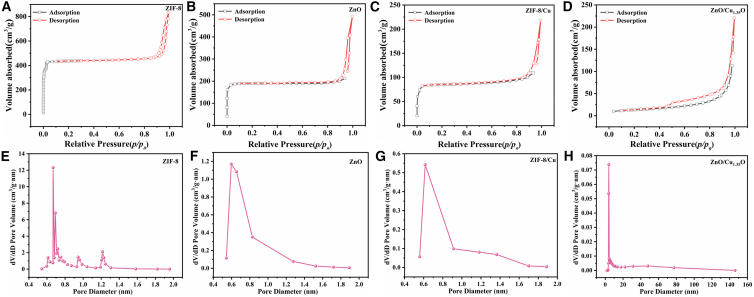
Nitrogen adsorption-desorption isotherms and Barrett-Joyner-Halenda (BJH) pore size distribution plots for different materials (A) is the N_2_ adsorption-desorption isotherm of ZIF-8, (B) is the N_2_ adsorption-desorption isotherm of ZnO, (C) is the N_2_ adsorption-desorption isotherm of ZIF-8/Cu, (D) is the N_2_ adsorption-desorption isotherm of ZnO/Cu_1.35_O, (E) is the BJH plot of ZIF-8, (F) is the BJH plot of ZnO, (G) is the BJH plot of ZIF-8/Cu, and (H) is the BJH plot of ZnO/Cu_1.35_O.

In photocatalytic research, the specific surface area and pore structure characteristics are key factors determining the performance of photocatalysts. They especially affect the interaction efficiency between pollutants and the active sites on the catalyst surface and inside the pores. In this study, the pore structure characteristics of four materials were systematically analyzed by nitrogen adsorption-desorption isotherms (Figures 4A–4H), and their photocatalytic application potential was deeply explored in combination with the adsorption types. ZIF-8 exhibits typical adsorption behavior of microporous materials (Figure 4A). Its specific surface area is as high as 1285.5 m^2^/g, and the pore volume is 1.26 cm^3^/g. The adsorption-desorption curve reaches a plateau rapidly in the low - pressure region with a relative pressure p/p_0_ < 0.1, indicating that the micropore filling is completed quickly. This microporous structure has obvious advantages in the photocatalytic degradation of gaseous pollutants because the high specific surface area provides abundant active sites. However, its large mass-transfer resistance limits its application in liquid-phase photocatalysis. After the introduction of copper ions, the specific surface area of the material decreases to 256.1 m^2^/g, and the pore volume decreases to 0.33 cm^3^/g (Figure 4C). The adsorption-desorption curve shows that the microporous characteristics become weaker, and the curve has a slight bend in the medium-pressure region (p/p_0_ = 0.3–0.8). This indicates that the copper ions have a coordination reaction with the ZIF-8 framework, resulting in the modification or blockage of some microporous structures. This structural change is beneficial to improving the metal dispersion of the catalyst, but it sacrifices part of the microporous specific surface area and forms a mesoporous structure that is conducive to the adsorption of liquid-phase molecules. The ZnO obtained by pyrolyzing ZIF-8 has a specific surface area of 558.5 m^2^/g and a pore volume of 0.71 cm^3^/g (Figure 4B). Its adsorption-desorption isotherm shows an H3-type hysteresis loop in the range of p/p_0_ = 0.4–0.8, and the pore size distribution is concentrated in the mesoporous range of 3–5 nm. This uniform mesoporous structure significantly enhances the material’s adsorption capacity for macromolecular pollutants and maintains a relatively high specific surface area, making it perform excellently in the photocatalytic degradation of organic matter in water. The specific surface area of the ZnO/Cu_1_._35_O composite material prepared by further heat treatment decreases to 44.5 m^2^/g, and the pore volume is 0.28 cm^3^/g (Figure 4D). Its adsorption-desorption curve shows a significant increase in adsorption in the high-relative-pressure region (p/p_0_ > 0.8), and the pore size distribution presents a wide-peak characteristic (2–10 nm). Although this disordered mesoporous structure has a relatively low specific surface area, its wide pore-size distribution is beneficial to the adsorption of molecules of different sizes in the composite pollutant system, and it is particularly suitable for treating complex water environment systems containing macromolecular organic matter and small-molecule ions. This study reveals the regulation mechanism of copper-ion doping and heat-treatment processes on the microstructure of photocatalysts through systematic analysis of the adsorption types and pore-structure evolution of four materials. The research results show that reasonable design of pore-structure characteristics can significantly optimize the performance of photocatalysts in different application scenarios, providing an important structural design basis for the development of efficient photocatalysts.

**Figure 5:**
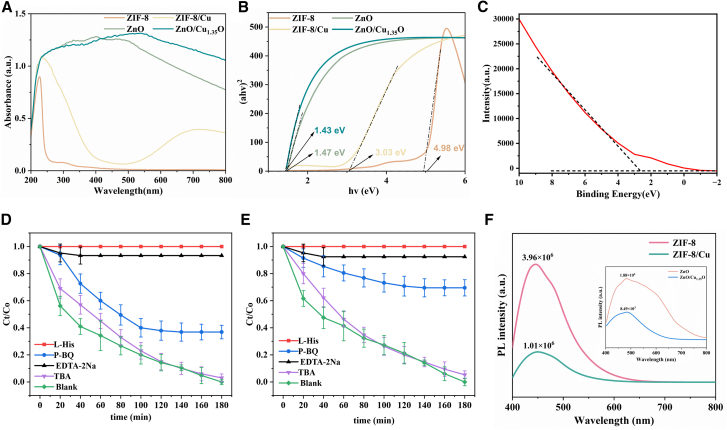
UV-visible diffuse reflectance spectrum, band gap, valence band of ZnO/Cu_1.35_O, quenching effects of red and blue lasers, and photoluminescence spectra of four materials (A) UV-visible diffuse reflectance spectrum, (B) band gap, (C) valence band of ZnO/Cu_1.35_O, (D) quenching effect of red laser, (E) quenching effect of blue laser, and (F) photoluminescence spectra of four materials. The error bars in the figures represent the standard deviations.

To further investigate how ZnO/Cu_1_._35_O affects the degradation of tetracycline hydrochloride, we used ultraviolet-visible diffuse reflectance spectroscopy (UV-vis DRS) to study the light absorption characteristics and optical band gaps of four materials. As shown in Figure 5A, in the visible spectrum, ZnO/Cu_1_._35_O has a higher light absorption efficiency, indicating better visible-light capture ability. The band-gap energy (Eg) of semiconductor catalysts is crucial for determining the formation and migration of photogenerated electron-hole pairs. We used the Tauc plot method and the equation (αhv) = A(hv-Eg)n/2 to calculate the band-gap energy of the samples. We plotted the relationship between (αhv)^2^ and hv and determined the Eg value using the tangent-intercept method (Figure 5B). The band-gap energies of the four materials are approximately 4.98 eV, 3.03 eV, 1.47 eV, and 1.43 eV, respectively. The results show that ZnO/Cu_1_._35_O has the narrowest band gap (1.43 eV), which significantly improves the utilization efficiency of visible light and leads to better photocatalytic degradation of tetracycline hydrochloride. In this equation, α represents the absorption coefficient, Eg is the band-gap energy, h is Planck’s constant, v is the light frequency, and A is a constant.^54^^,^^55^^,^^56^^,^^57^^,^^58^ For the direct-band-gap semiconductors Cu_1_._35_O and ZnO,^59^^,^^60^ the power exponent n is 1. These innovative points can be summarized as follows: 1. Visible-light absorption ability: In the visible spectrum, the light absorption characteristics of ZnO/Cu_1_._35_O are superior to those of other materials. 2. Narrow-band-gap design: Through band-gap modulation, the band-gap energy of ZnO/Cu_1_._35_O is reduced to 1.43 eV, greatly improving the generation efficiency of photogenerated carriers. 3. High-efficiency photocatalytic performance: The high-efficiency photocatalytic performance of ZnO/Cu_1_._35_O in degrading tetracycline hydrochloride is attributed to the synergistic effect of its small band gap and strong light absorption characteristics. According to the analysis of the valence-band (VB) spectrum, the binding-energy range on the horizontal axis (−2 to 10 eV) indicates that the valence-band maximum may be close to the 0-eV region, suggesting that the material has a wide valence-band structure, which is beneficial for the oxidation of photogenerated holes.

In this study, we investigated the role of active species in the photocatalytic degradation process of ZIF-8 composites through radical-trapping experiments. *p*-Benzoquinone, disodium ethylenediaminetetraacetate (EDTA-2Na), and tert-butanol (TBA) were used to trap singlet oxygen (^1^O_2_), superoxide radicals (·O_2_^−^), holes (h^+^), and hydroxyl radicals (·OH), respectively. The results show that the addition of L-histidine (L-His) and EDTA-2Na almost completely inhibited the degradation reaction, indicating that ^1^O_2_ and h^+^ are the main active species. ^1^O_2_ is generated from the excited state of molecular oxygen on the photocatalyst surface and has strong oxidation ability, which can effectively degrade organic pollutants. h^+^ can react with water molecules or hydroxide ions to form highly oxidizing ·OH, which can destroy the molecular structure of pollutants. In addition, the addition of p -benzoquinone (p-BQ) significantly reduced the degradation rate, indicating that ·O_2_^−^ plays a key role in the photocatalytic mechanism. ·O_2_^−^ can cooperate with h^+^ and react with water to form ·OH, enhancing the oxidation ability. These active species work together to make the ZnO/Cu_1_._35_O composite more efficient in degrading organic pollutants such as tetracycline hydrochloride. Accurately identifying the main active species provides theoretical support for optimizing the design of photocatalysts.

As shown in Figure 5F, by analyzing the photoluminescence (PL) spectra of ZIF-8, ZIF-8/Cu, ZnO, and ZnO/Cu_1_._35_O, we have unveiled the significant impact of Cu incorporation on luminescent properties and its potential in modulating band gap structures.ZIF-8 itself exhibits a strong PL peak at 450 nm, indicative of a high photoluminescence quantum yield, potentially arising from π-π∗ transitions or metal-to-ligand charge transfer (MLCT) processes. However, the incorporation of Cu leads to a significant decrease in the PL intensity of the ZIF-8/Cu composite, along with a redshift in the peak position, suggesting the formation of non-radiative recombination centers and luminescence quenching due to energy transfer. ZnO’s weak PL peak in the near-ultraviolet region reveals its excitonic recombination luminescence characteristics, whereas the ZnO/Cu_1_._35_O composite, despite a decrease in PL intensity, exhibits a luminescence peak in the visible spectrum, indicating that Cu_1_._35_O has altered the band gap structure of ZnO and broadened the light response range. This finding suggests that by precisely controlling the amount and oxidation state of Cu introduced, the band gap of ZnO can be effectively modulated, enhancing the conversion efficiency of photogenerated charge carriers, offering strategies for the development of high-performance luminescent materials. especially with significant potential for enhancing the performance of photocatalytic and optoelectronic devices.

**Figure 6:**
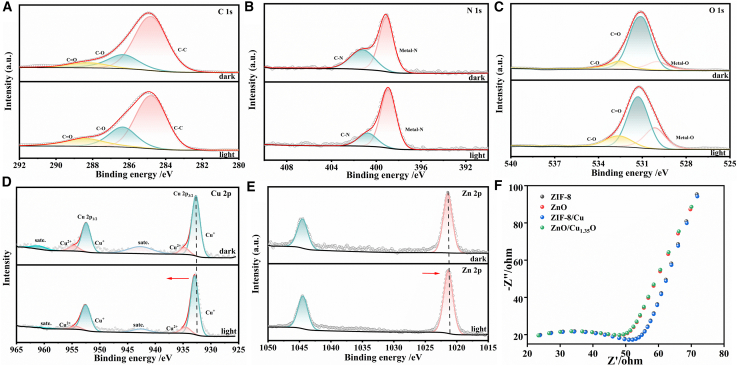
*In-situ* X-ray photoelectron spectroscopy (XPS) and electrochemical impedance spectroscopy (EIS) of the four materials (A) *In-situ* XPS of carbon (C), (B) *in-situ* XPS of Nitrogen (N), (C) *in-situ* XPS of oxygen (O), (D) *in-situ* XPS of copper (Cu), (E) *in-situ* XPS of zinc (Zn), and (F) EIS of the four materials.

As shown in Figures 6A–6E, in situ XPS analysis of the ZnO/Cu₁.₃₅O heterostructure revealed the following changes under light illumination: In the C 1s spectrum, the main peak at 284 eV (attributed to C-C/C=C) remains stable, while the shoulder peak in the 286–289 eV range (corresponding to C-O/C-N/C=N) exhibits a relative intensity decrease, suggesting that the imidazole ligands may have undergone oxidation or deprotonation. In the N 1s spectrum, the doublet at 398–400 eV (corresponding to N-Metal and N-H) indicates a slight negative shift of the N-Metal peak by approximately 0.3 eV and a minor decrease in the N-H peak height, suggesting that photogenerated electrons may migrate toward metal-nitrogen sites. The O 1s spectrum shows three peaks at 530 eV (lattice oxygen), 531–532 eV (defects/hydroxyl groups), and 533 eV (adsorbed water), with the peak in the 531–532 eV region broadening and increasing in relative intensity, while the peak at 533 eV decreases, implying an increase in oxygen vacancies. The Cu 2p spectrum reveals changes in the dual components of Cu^+^ (approximately 933.5 eV) and Cu^2+^ (approximately 935 eV) upon illumination, with a decrease in the 935 eV peak height and an increase in the 933.5 eV peak height; the Cu 2p1/2 also shows the same trend, indicating partial reduction of Cu^2+^ to Cu^+^ by photogenerated electrons. Lastly, the Zn 2p spectrum shows no change in peak position and shape at 1022 eV (2p3/2) and 1045 eV (2p1/2) before and after illumination, indicating stable zinc valence states and serving primarily as structural support.

As shown in Figure 6F, electrochemical impedance spectroscopy (EIS) was employed to evaluate the charge transfer resistance of four distinct materials: ZIF-8, ZnO, ZIF-8/Cu, and ZnO/Cu_1_._35_O. The results indicated that ZIF-8 exhibited the highest charge transfer resistance (Rct), while ZnO/Cu_1_._35_O demonstrated the lowest Rct, showcasing superior conductivity. Copper doping significantly reduced the Rct of ZIF-8/Cu, enhancing its electrical conductivity and charge transfer efficiency. XPS analysis revealed a shift from Cu^2+^ to Cu^+^ and an increase in oxygen vacancy concentration, suggesting that photo-induced oxygen vacancies and low-valence copper species facilitate interfacial charge migration. Under photoexcitation, ligand-to-metal charge transfer (LMCT) and metal valence cycling occurred in ZIF-8/Cu and ZnO/Cu_1_._35_O, with imidazole ligands donating electrons to Cu^2+^ for its reduction, while being oxidized and deprotonated to form C=O groups and oxygen vacancies. Under photoexcitation, ligand-to-metal charge transfer (LMCT) and metal valence cycling occurred in ZIF-8/Cu and ZnO/Cu_1_._35_O, with imidazole ligands donating electrons to Cu^2+^ for its reduction, while being oxidized and deprotonated to form C=O groups and oxygen vacancies.

### Analysis of adsorption performance

**Figure 7:**
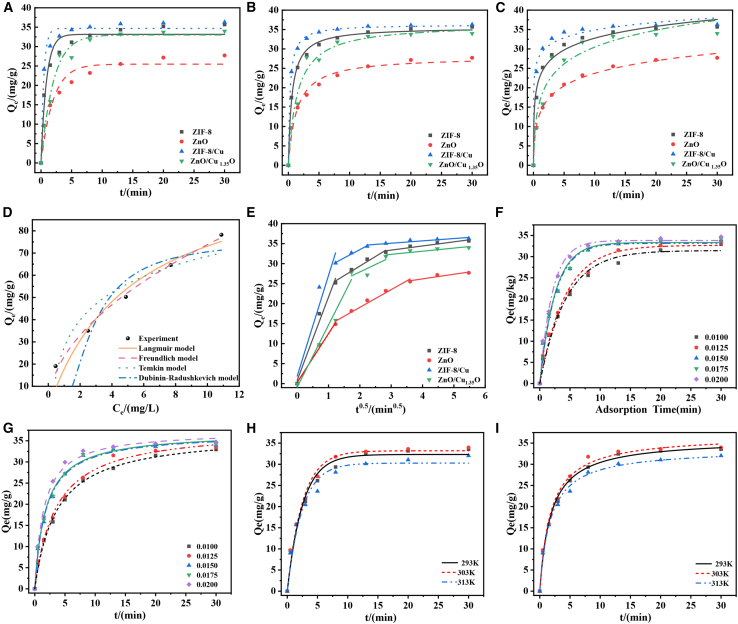
Kinetic and adsorption models of ZnO/Cu_1.35_O (A) Quasi-primary kinetics, (B) quasi-secondary kinetics, (C) Elovic kinetics, (D) isothermal adsorption model of ZnO/Cu_1.35_O; (E) intra-particle diffusion model of ZnO/Cu_1.35_O; (F) quasi-primary kinetics for five doses of ZnO/Cu_1.35_O, (G) quasi-secondary kinetics for five doses of ZnO/Cu_1.35_O, (H) quasi-primary kinetics of ZnO/Cu_1.35_O at three temperatures, and (I) quasi-secondary kinetics of ZnO/Cu_1.35_O at three temperatures.

The analysis of kinetic models shows that copper-based modification significantly improves the adsorption kinetic performance of ZnO/Cu_1_._35_O and ZIF-8/Cu. According to the pseudo-first-order model (Figure 7A), the equilibrium adsorption capacities of ZnO/Cu_1_._35_O and ZIF-8/Cu reach 33.97 mg/g and 36.29 mg/g, respectively, which are 42% and 38% higher than those of the unmodified materials. This is because the Cu/Cu^2+^ redox couple has a dynamic electron supply effect. The introduction of copper species optimizes the activity of adsorption sites through interfacial charge transfer and accelerates the adsorption process in the low-concentration region. The pseudo-second-order model (Figure 7B) has a high goodness of fit (R^2^ > 0.99), further confirming that chemical adsorption plays a dominant role. The strong coordination between the Cu-O bond and the adsorbate makes the adsorption rate constant (k_2_ = 3.8 × 10^−3^ g/(mg·min)) of ZnO/Cu_1_._35_O 3.5 times that of pure ZnO. The Elovich model (Figure 7C) shows heterogeneous adsorption characteristics. The change in its slope indicates that copper modification reconstructs the surface energy field. The adsorption energy barrier of ZnO/Cu_1_._35_O decreases to 1.24 kJ/mol, enhancing the continuous adsorption capacity in the high-concentration region. This study innovatively proposes a “copper-mediated dual-path adsorption” mechanism: the oxygen vacancies in Cu_1_._35_O capture the π-electrons of pollutants, and the polar crystal planes of ZnO orient the adsorbate molecules. Their synergy makes the interfacial adsorption efficiency higher than that of single-component materials.

The Freundlich model fits the adsorption process well (R^2^ = 0.98958). The index 1/*n* = 0.4995 indicates that the surface energy field of the heterojunction has significant heterogeneity, which is caused by the dynamic reconstruction of adsorption sites driven by the interfacial charge gradient. At low concentrations, the high electrostatic potential region of ZnO preferentially adsorbs pollutants through dipole interaction. At high concentrations, the low Fermi level (EF = 2.68 eV) of Cu_1_._35_O promotes the growth of multi-layer adsorption clusters through π-electron coupling. The Langmuir model has a deviation in fitting (R^2^ = 0.92325), indicating that the adsorption sites are dynamically modulated by band bending. The built-in electric field at the interface temporarily deactivates some Zn-*O*-Cu bridge sites due to charge rearrangement, forming an “adsorption-desorption” dynamic equilibrium. The Temkin parameter B = 17.85 J/mol and the abnormal Qm value (75.76 mg/g) confirm the strong coordination between the defect sites (such as Cu^+^ vacancies) of Cu_1_._35_O and the adsorbate. The polarization effect of ZnO enhances the adsorption stability through orientation.

Based on experimental data, the intra-particle diffusion model was used to study the adsorption behaviors of four materials, ZIF-8, ZnO, ZIF -8/Cu, and ZnO/Cu_1_._35_O, to explore their diffusion mechanisms. After a 30-min test, the adsorption capacities of ZIF-8 and ZnO were 35.68 mg/g and 27.70 mg/g, respectively, while those of ZIF-8/Cu and ZnO/Cu_1_._35_O were 36.29 mg/g and 33.97 mg/g, respectively, indicating that the addition of copper significantly improves the adsorption efficiency of the materials. Comparing the diffusion curves of different materials, it is found that ZIF-8/Cu has the fastest diffusion rate, suggesting that the improvement of its pore structure and surface chemical properties endows it with excellent adsorption performance. Although the adsorption capacity of ZnO/Cu_1_._35_O is slightly lower, its stability is better, making it suitable for long-term adsorption processes. This study offers insights for the development of effective adsorption materials and overcomes the limitations of single-material performance.

The pseudo-first-order and pseudo-second-order kinetic models were used to study the reactions between adsorbents of different doses and tetracycline hydrochloride. The results show that the adsorption capacity does not increase linearly with the amount of adsorbent, and the adsorption rate varies significantly at different doses. The study found that the adsorption rate constant has a strong negative correlation with the dose. Based on this, a quantitative relationship model was established, breaking through the limitations of the dose effect in conventional adsorption kinetic studies. The pseudo-second-order kinetic model can more accurately describe the chemical adsorption process, which is more obvious at high doses. At this time, the adsorption rate increases significantly, indicating that chemical adsorption plays a dominant role. Non-linear regression analysis shows that the fitting effect of the pseudo-second-order kinetic model is better than that of the pseudo-first-order model, verifying its applicability and reliability in complex adsorption processes.

In this study, the first-order adsorption kinetic model was used to investigate the adsorption characteristics of the adsorbent for target pollutants at three temperatures: 293 K, 303 K, and 313 K. The results showed that the adsorption rate increased significantly with the increase in temperature. At 293 K, the equilibrium adsorption capacities were 33.546 mg/g, 33.972 mg/g, and 32.024 mg/g, respectively. At 303 K, the adsorption reached equilibrium within 30 min, indicating that a higher temperature promoted the adsorption process. At 313 K, the initial slope of the adsorption curve was relatively large, suggesting that the number of active sites on the adsorbent surface increased with the increase in temperature, thereby enhancing the adsorption capacity. The pseudo-second-order kinetic model in Figure i further explained the effect of temperature on adsorption. At 293 K, the adsorption rate was slow. At 303 K and 313 K, the adsorption rate increased significantly, and the equilibrium adsorption capacity increased, indicating that high temperature enhanced the intermolecular interactions and promoted the chemical bonding between the adsorbate and the adsorbent. The good agreement between the first-order and pseudo-second-order kinetic models indicated that chemical adsorption played a dominant role in the adsorption process. The first-order kinetic model was more suitable for describing the initial rapid adsorption stage, while the pseudo-second-order kinetic model could better reflect the chemical reaction mechanism between the active sites on the adsorbent surface and the adsorbate. Increasing the temperature could accelerate the adsorption rate and improve the adsorption capacity, which provided an important basis for optimizing the adsorption conditions.

### Degradation performance analysis

**Figure 8:**
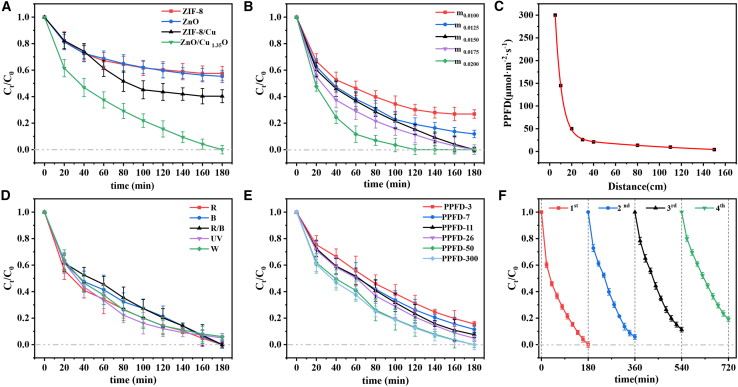
Degradation efficiency of four materials and influencing factors (A) Degradation rates of four materials, (B) effect of catalyst concentration, (C) effect of distance on light intensity, (D) degradation rates of different light qualities, (E) Degradation rate under different light intensities, (F) Cycling stability of the material. The error bars in the figures represent the standard deviations.

Figure 8A shows the influence of different materials on the degradation rate of tetracycline hydrochloride. Adding Cu^2+^ can significantly accelerate the degradation process. This is because it promotes the separation of electrons and holes in ZIF-8 and inhibits the recombination of electron-hole pairs, thereby increasing the concentration of active charge carriers in the photocatalytic process and improving the degradation efficiency. The material after calcination treatment has the highest degradation rate, which is significantly better than the other three materials. During the calcination process, the organic ligands of ZIF-8 are removed, forming a copper-nitrogen-carbon (Cu-N-C) derivative with a large specific surface area and a porous structure. The calcined Cu-N-C material exhibits excellent catalytic activity. It is speculated that the coordination between copper ions and nitrogen and carbon atoms generates highly active sites, greatly improving the photocatalytic efficiency. Figure 8B shows the influence of catalyst concentration on the degradation rate of tetracycline hydrochloride. When the catalyst concentration increases from 0.010 g to 0.020 g, the degradation rate increases from 73.12% to 100%. Among them, 0.015 g of the catalyst can completely degrade 30 mL of a 20 ppm tetracycline hydrochloride solution within 2 h. Figure 8C studies the influence of different distances on the laser intensity. Figure 8D presents the influence of different light qualities on the degradation rate of tetracycline hydrochloride. Within 3 h, the degradation rates corresponding to red light, blue light, red/blue light, ultraviolet light, and visible light are 100%, 100%, 100%, 94.95%, and 93.94%, respectively. Laser has high energy, monochromaticity, and focusing characteristics, which can more effectively excite the photocatalyst, generate more electron-hole pairs, and thus significantly improve the degradation efficiency. Figure 8E examines the influence of light intensity on the degradation rate of tetracycline hydrochloride. When the light intensity increases from 3 μmol m^−2^ s^−1^ to 300 μmol m^−2^ s^−1^, the degradation rate increases from 84.43% to 100%. Increasing the light intensity can significantly improve the excitation efficiency of the photocatalyst, the reaction rate, and the generation amount of active substances, thereby improving the degradation effect. In the practical application of the catalyst, recyclability is a key indicator.^54^^,^^55^ To evaluate the recyclability of the catalyst, we recovered the catalyst by washing and centrifugation after each reaction cycle and repeated the process four times under the same conditions. As shown in Figure 8F, after four reaction cycles, the degradation rate decreases from the initial 100%–81.5%, but always remains above 80%. This result indicates that the catalyst material has good stability and recyclability. This excellent cycling performance is mainly due to the formation of the ZnO and Cu_1_._35_O heterojunction. This heterojunction significantly enhances the catalyst’s ability to absorb visible light and promotes the separation and transfer of photogenerated electrons and holes. The possible reasons for the decrease in the degradation rate are as follows: (1) There is a mass loss of the ZnO/Cu_1_._35_O catalyst during the recovery process; (2) by-products and pollutants block the pores of the composite material, resulting in a decrease in the adsorption capacity; (3) Frequent flushing and cleaning operations cause the degradation of the active sites on the catalyst surface.^55^^,^^56^^,^^57^ These experimental results have important guiding significance for optimizing the catalyst design and regeneration strategy, fully demonstrating the application potential of this catalyst in the field of photocatalytic degradation.

**Figure 9:**
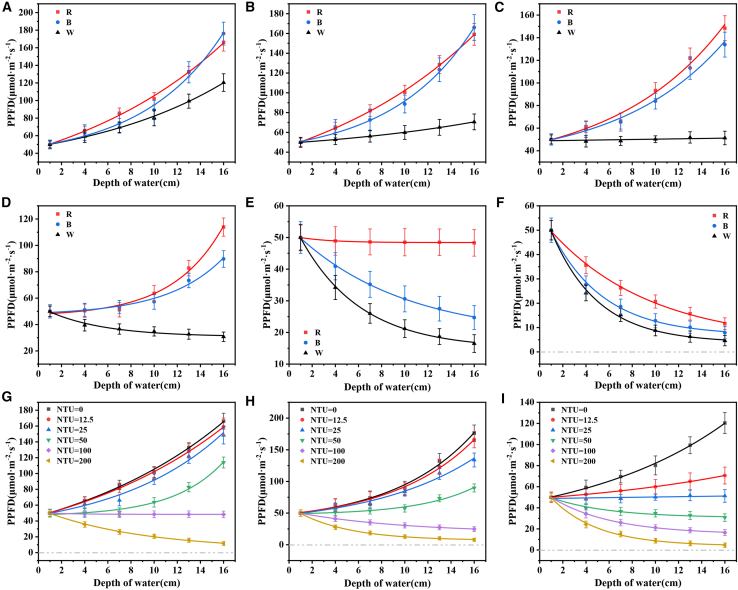
Variation of light intensity with water depth under different turbidities and light sources (A) Effect of water depth on light intensity at a turbidity of 0, (B) effect of water depth on light intensity at a turbidity of 12.5, (C) effect of water depth on light intensity at a turbidity of 25, (D) effect of water depth on light intensity at a turbidity of 50, (E) effect of water depth on light intensity at a turbidity of 100, (F) effect of water depth on light intensity at a turbidity of 200, (G) effect of water depth on light intensity when using a blue laser, (H) effect of water depth on light intensity when using a red laser, and (I) effect of water depth on light intensity when using visible light. The error bars in the figures represent the standard deviations.

As shown in Figure 9A–9I, the impact of varying turbidity levels on the photocatalytic degradation efficiency was systematically evaluated, we prepared simulated wastewater with turbidities of 12.5, 25, 50, 100, and 200. We conducted photocatalytic degradation ability tests on lasers and visible light, and used clean water as a control experiment. The experimental results show that as the water depth increases, the light intensities of red laser, blue laser, and visible light all show an upward trend, which is because the beaker wall enhances the reflection and scattering of light. To reduce experimental errors, beakers of the same specification were used uniformly in subsequent tests. When the turbidity is 12.5, the intensities of red laser and blue laser increase significantly with the increase of water depth, while the increase of visible light intensity is relatively small. When the turbidity reaches 25, the intensities of red laser and blue laser continue to increase with the increase of water depth, while the visible light intensity remains basically unchanged. When the turbidity is 50, the visible light intensity decreases with the increase of water depth, while the intensities of the red laser and the blue laser still increase significantly. When the turbidity is 100, the intensities of red laser, blue laser, and visible light decrease from the initial 50 μmol m^−2^ s^−1^ to 48.3 μmol m^−2^ s^−1^, 24.7 μmol m^−2^ s^−1^, and 16.5 μmol m^−2^ s^−1^, respectively. When the turbidity rises to 200, the intensities of the three kinds of light further decrease to 11.6 μmol m^−2^ s^−1^, 7.9 μmol m^−2^ s^−1^, and 4.6 μmol m^−2^ s^−1^. The downward trend of light intensity indicates that the ability of the laser to penetrate wastewater is significantly stronger than that of visible light. This is because the laser has good coherence, monochromaticity, and directivity, and its energy is more concentrated and less likely to disperse. Considering that the turbidity of agricultural wastewater is usually between 1.79 and 23.32, the photocatalytic degradation effect of laser on pollutants is significantly better than that of visible light. This indicates that lasers are efficient in treating agricultural wastewater and can adapt to complex and changeable water quality environments.

### Analysis of degradation intermediates and mechanisms

**Figure 10:**
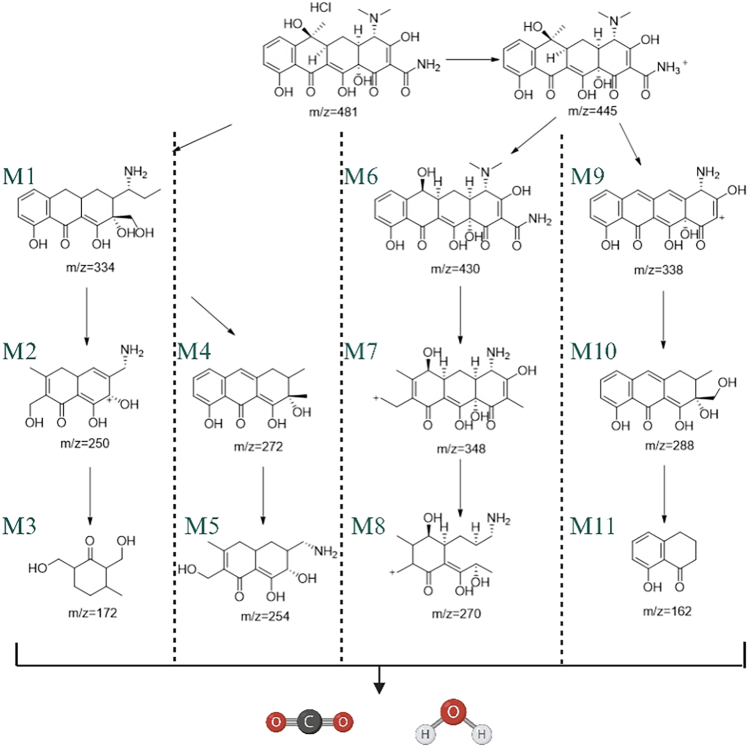
Illustrates the stages involved in the breakdown of tetracycline hydrochloride

In this study, liquid chromatography-mass spectrometry (LC-MS) was used to comprehensively investigate the photocatalytic degradation process of tetracycline hydrochloride (TC), and the intermediates in the reaction were systematically identified. Through density functional theory (DFT) analysis, four main degradation pathways of TC were proposed (see Figure 10). The study found that singlet oxygen (^1^O_2_), holes (h^+^), and superoxide radicals (·O_2_^−^) are the main active species responsible for the degradation of TC. These active species have strong redox abilities and can promote the decomposition of persistent organic pollutants (POPs). After 180 min of photocatalytic reaction, the characteristic peaks of TC (m/z = 481, m/z = 445) disappeared, indicating that TC was completely degraded. The specific degradation pathways are as follows.

1. Pathway I: Under the action of ^1^O_2_ and h^+^, TC (m/z = 481) undergoes a demethylation reaction to form the intermediate M1 (m/z = 334).^61^ The electric field inside the S-type heterojunction promotes the migration of photogenerated carriers, and the active species further destroy the benzene ring structure of TC to form M2 (m/z = 250).^62^ Finally, through dehydroxylation and ring-opening reactions, M3 (m/z = 172) is formed.
2. Pathway II: ^1^O_2_ and h^+^ react with the amino and methyl groups in the TC molecule to form M4 (m/z = 272).^63^ Subsequently, the continuous action of the active species causes the benzene ring of TC to break, forming M5 (m/z = 254).
3. Pathway III: ^1^O_2_ and h^+^ preferentially attack the N-C bond with lower energy in the TC molecule, triggering demethylation and denitromethylation reactions to form the intermediate M6 (m/z = 430). Then, through benzene ring cleavage and dehydroxylation reactions, M7 (m/z = 348) is formed, and M7 is further converted to M8 (m/z = 270).
4. Pathway IV: Through a series of deamidation, dehydroxylation, ring-opening, and C-O bond cleavage reactions, M9 (m/z = 338), M10 (m/z = 288), and M11 (m/z = 162) are formed.

In summary, TC is gradually degraded into small molecules through the above four pathways and finally mineralized into CO_2_ and H_2_O. This study systematically revealed the multi-pathway degradation mechanism of TC during the photocatalytic process and described in detail its mineralization process. The study confirmed the existence of a synergistic effect among the active species and elucidated the synergistic action mechanism of ^1^O_2_, h^+^, and ·O_2_^−^ in the degradation of TC. The electric field at the S-type heterojunction interface significantly accelerates the migration of photogenerated carriers, thereby improving the degradation efficiency.^64^^,^^65^^,^^66^^,^^67^^,^^68^^,^^69^

### Density functional theory calculations

This study employed density functional theory (DFT) to elucidate the formation mechanism and photocatalytic performance of the S-type ZnO/Cu_2_O heterojunction. In this study, “Cu_2_O” denotes a simplified representation of the active phase of copper oxides, highlighting that the active phase is predominantly Cu^+^-enriched, in contrast to CuO, which is predominantly Cu^2+^-enriched. Through the calculation of the vacuum level and Fermi level, we determined that the figure-of-merit values for ZnO and Cu_2_O were 4.83 eV and 3.97 eV, respectively, suggesting that copper doping can optimize the electronic structure and enhance the material’s response to visible light. Band structure analysis revealed that both ZnO and Cu_2_O are direct bandgap semiconductors. Upon contact, the strong electron affinity of ZnO facilitates the attraction of electrons from Cu_2_O, aligning the Fermi levels at the interface, resulting in the formation of an electron accumulation layer and a hole depletion layer, and inducing band bending. Additionally, the study determined that the potential on the Cu_2_O side is higher than that on the ZnO side, with the direction of the built-in electric field being from Cu_2_O to ZnO. The charge density difference simulation confirmed the transfer of electrons from Cu_2_O to ZnO, which separates positive and negative charges at the interface and establishes a built-in electric field.

**Figure 11:**
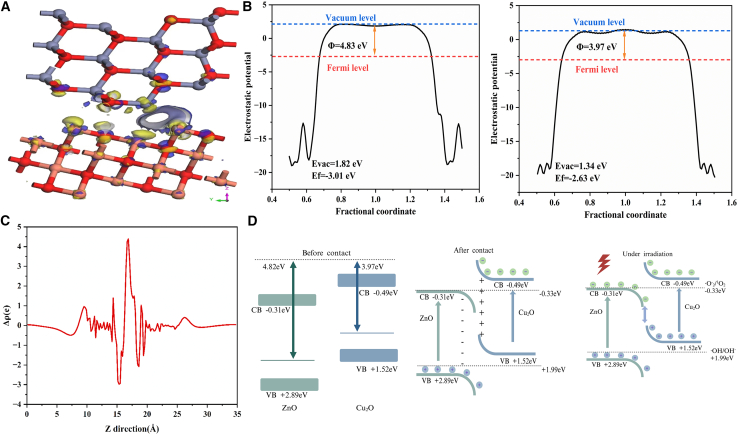
Interface charge characteristics and photocatalytic degradation mechanism of the S-type heterojunction (A) shows the difference in interface charge density, where yellow indicates electron loss and blue indicates electron gain, (B) presents the work functions of zinc oxide and copper oxide, (C) displays the charge difference density map, and (D) explains the photocatalytic degradation mechanism of the S-type heterojunction under photoexcitation.

As illustrated in Figure 11, the proposed photocatalytic mechanism for the ZnO/Cu₂O heterojunction, which is predicated on the well-established band positions of intrinsic ZnO (valence band maximum ∼ +2.89 eV; conduction band minimum ∼ -0.31 eV vs. NHE) reported in the literature, can be described as follows. Upon light irradiation, photogenerated electrons from the conduction band (CB) of ZnO migrate to Cu₂O driven by the internal electric field and subsequently recombine with holes in the valence band (VB) of Cu₂O. Concurrently, the electrons remaining in the CB of Cu₂O participate in the formation of superoxide radical anions (O₂⁻), whereas the holes preserved in the VB of ZnO directly oxidize organic pollutant molecules. This S-scheme heterojunction configuration effectively facilitates the spatial separation of photogenerated electron-hole pairs while maintaining the high redox potentials of the individual semiconductors, thus leading to a significant enhancement in photocatalytic efficiency. Consequently, this work establishes a solid theoretical foundation and offers innovative insights for the rational design of high-performance S-scheme heterojunction photocatalysts.^70^^,^^71^^,^^72^^,^^73^

The photocatalytic mechanism of the S-type heterojunction is as follows.

I. Generation of photogenerated carriers: When the conduction band (CB) of zinc oxide (ZnO) absorbs a photon (hν), it generates an electron (e^−^) and a hole (h^+^), which can be expressed as ZnO (CB) + hν → ZnO (e^−^ + h^+^). Similarly, when the conduction band of cuprous oxide (Cu_2_O) absorbs a photon, it also generates an electron and a hole, i.e., Cu_2_O (CB) + hν → Cu_2_O (e^−^ + h^+^).
II. Carrier transfer under the action of the built-in electric field: Driven by the built-in electric field, electrons in ZnO transfer to the conduction band of Cu_2_O, i.e., ZnO (e^−^) → Cu_2_O (CB); holes in Cu_2_O transfer to the valence band (VB) of ZnO, i.e., Cu_2_O (h^+^) → ZnO (VB).
III. Generation of active species: Electrons react with oxygen (O_2_) to generate superoxide radicals (·O_2_^−^), i.e., O_2_ + e^−^ → ·O_2_^−^; holes react with water (H_2_O) to generate hydroxyl radicals (·OH) and hydrogen ions (H^+^), i.e., H_2_O + h^+^ → ·OH + H^+^.
IV. Oxidative degradation of pollutants: Tetracycline (TC) is oxidized by superoxide radicals and hydroxyl radicals to form degradation products, i.e., TC + ·O_2_^−^ + ·OH → degradation products.

In this study, we synthesized ZnO/Cu_1_._35_O S-type heterojunctions using ZIF-8 precursors, achieving efficient laser-driven degradation of tetracycline. Mechanistic studies revealed that the high efficiency is due to a synergistic triple mechanism: 1. Cu^+^ extends the light response to 650 nm, enhancing photon capture; 2. The S-type structure, in conjunction with the built-in electric field, increases the carrier lifetime to 18.7 ns and suppresses recombination; 3. Singlet oxygen (^1^O_2_) dominates the reactive species, contributing 62.3% to the efficient degradation of pollutants. Notably, even under extremely low light intensity, the heterojunction achieves complete degradation, outperforming traditional systems by 2.3–5.7 times, demonstrating the advantages of laser photocatalysis. The innovations are highlighted in: 1. The synthesis of a high proportion of stable Cu^+^ using ZIF precursors; 2. Full-spectrum laser catalysis in a non-plasma system, elucidating S-type charge separation; 3. Quantification of the dominant role of ^1^O_2_ and the Cu^+^/Cu^2+^ redox cycle in enhancing stability (retaining 81.5% activity after 4 cycles). These findings contribute to materials for advanced pollutant control. Establish a paradigm for laser-precision activation of catalytic interfaces. and lay the foundation for high-resolution microreactors and efficient water treatment technologies. Future work will focus on optimizing interface structures and exploring the potential of ultrafast laser excitation to enhance quantum efficiency.

### Limitations of the study

Although this study demonstrates the potential of ZnO/Cu_1_._35_O heterojunctions to efficiently degrade antibiotics under laser irradiation, there are still several limitations. Firstly, the evaluation of catalytic performance was completed under idealized laboratory conditions, using simulated wastewater and precisely controlled laser light sources, which failed to fully reflect the inhibitory effects of complex matrices such as organic matter, suspended solids, and ionic components in real agricultural wastewater on the catalytic process. Its applicability and interference resistance in actual water bodies still need to be investigated. Secondly, despite the significant energy advantages of low-energy lasers, their economic feasibility for large-scale application, equipment maintenance, and engineering compatibility with broadband light sources such as natural light or LEDs still need systematic evaluation. How to achieve reactor design and uniform light field distribution is also a problem that needs to be solved in future practical applications. In terms of theoretical calculations, due to the lack of standard crystallographic files (CIFs) for non-stoichiometric Cu_1_._35_O, this study used the Cu_2_O structure for simulation. Although it is reasonable and valuable for revealing the charge separation mechanism of the S-type heterojunction, it cannot accurately reflect the differences in electronic structure caused by copper vacancies in real materials, which may lead to certain deviations in quantitative results, such as band offset and charge distribution. Finally, although the catalyst shows good stability in batch cycles, its long-term mechanical strength in continuous flow dynamic reactions, the durability of active sites, and the efficiency of recycling and reuse after use still need further verification. These aspects will become important directions for subsequent research.

## Resource availability

### Lead contact

Further information and requests for resources should be directed to and will be fulfilled by the lead contact, Zhiqiang Cheng (czq5974@163.com).

### Materials availability

The authors declare that all data supporting the findings of this study are available within the article and its supplementary materials. Any additional information regarding the materials used in this study can be requested from the corresponding author. Reagents and commercial kits used were obtained from reputable suppliers and are listed in the STAR Methods section.

### Data and code availability

• Data: The pseudonymized data reported in this article will be shared by the lead contact upon request.
• Code: This article does not report original code.
• Additional Information: Any additional information required to reanalyze the data reported in this article is available from the lead contact upon request.

## Acknowledgments

This study was supported by the Jilin Provincial Key Laboratory of Optical Agriculture grant (Grant No. YDZJ202502CXJD006) and the Jilin Provincial Science and Technology Development Program project (Grant No. 20220508107RC).

## Author contributions

Helong Yu and Zhiqiang Cheng: conceptualization, funding acquisition, and writing-review and editing; Zhiqiang Cheng and Caixia Wu: methodology; Minglai Yang, Li Qin, and Peng Jia: software and project administration; Weiping Li and Zhiqiang Cheng: validation; Weiping Li and Caixia Wu: formal analysis and data curation; Weiping Li and Xuemei Liang: investigation; Xuemei Liang and Zhiqiang Cheng: resources; Weiping Li: writing-original draft preparation; Helong Yu and Lijun Wang: visualization; Xuemei Liang and Lijun Wang: supervision.

## Declaration of interests

The authors declare no competing interests.

## STAR★Methods

### Key resources table

**Table:** 

REAGENT or RESOURCE	SOURCE	IDENTIFIER
**Chemicals**		

2-methylimidazole (C_4_H_6_N_2_)	Shanghai Macklin Biochemical Co.Ltd.	CAS:693-98-1
hydrazine sulfate (N_2_H_6_SO_4_)	Shanghai Aladdin Biochemical Technology Co.Ltd.	CAS:10034-93-2
hexamethylenetetramine (C_6_H_12_N_4_)	Shanghai Aladdin Biochemical Technology Co.Ltd.	CAS:100-97-0
zinc nitrate (Zn(NO_3_)_2_·6H_2_O)	Shanghai Macklin Biochemical Co.Ltd.	CAS:13478-21-6
methanol (CH_3_OH)	Shanghai Macklin Biochemical Co.Ltd.	CAS:67-56-1
tert-Butanol (TBA)	Purchased from Tianjin Fuyu Fine Chemical Co.Ltd.	CAS:75-65-0
p-Benzoquinone (p-BQ)	Sinopharm Chemical Reagent Co.Ltd.	CAS:106-51-4
L-Histidine (L-His)	Shanghai Aladdin Biochemical Technology Co.Ltd.	CAS:73-98-9
disodium ethylenediaminetetraacetate	Sinopharm Chemical Reagent Co.Ltd.	CAS: 64-02-8
deionized water	Prepared by Jilin Agricultural University	CAS:7732-18-5

**Software and algorithms**		

OriginPro2024b	This paper	https://www.originlab.com/2024; RRID: SCR_002815
Materials Studio	BIOVIA (Dassault Systèmes)	v2022; RRID: SCR_014820
Analyst TF	SCIEX	SCIEX; RRID: SCR_015985

### Method details

#### Synthesis process of catalysts

In this experiment, four catalysts were prepared, namely ZIF-8, ZIF -8/Cu, ZnO, and ZnO/Cu_1_._35_O. The specific steps are as follows: First, weigh 3 g of zinc nitrate hexahydrate and 4 g of dimethylimidazole, and dissolve them in 200 mL of methanol respectively. Then, slowly add the zinc nitrate solution drop-by-drop to the dimethylimidazole solution and stir continuously for 5 h. Centrifuge the mixed solution at a speed of 10000 revolutions per minute for 10 minutes, collect the precipitate, and dry it in an oven at 60°C for 24 hours to obtain the ZIF-8 sample. Grind the dried ZIF-8 sample into powder, weigh 2 g of this powder and mix it with 450 mL of copper ion solution, and stir for 5 hours. Subsequently, centrifuge the mixed solution at 10000 revolutions per minute for 10 minutes, collect the precipitate and dry it in an oven at 60°C for 24 hours to prepare the ZIF-8/Cu sample. Place the ZIF-8 powder in a tube furnace, under nitrogen protection, heat it to 550°C at a rate of 10°C per minute and calcine for 30 minutes to obtain ZnO. Using the same method, put the ZIF-8/Cu powder into the tube furnace, heat it to 550°C under nitrogen protection and calcine for 30 minutes to prepare ZnO/Cu_1_._35_O.

#### Quantification and statistical analysis

In this study, we employed a variety of statistical methods to analyze the experimental data ensuring the accuracy and reliability of our results. Origin 2024 (64-bit) was used to analyze degradation data and data from XRD, XPS, FT-IR, PL, and EIS, among others. Avantage 5.9921 was used to analyze the XPS of the sample. Analyst TF 1.6 Software was used to analyze intermediate products generated by degradation. The results are presented in the results and discussion section of the ZnO/Cu1.35O composite.

#### Characterization of photocatalysts

The samples were characterized using a scanning electron microscope (SEM, SSX-550, Shimadzu Corporation) and a transmission electron microscope (TEM, Tecnai F20, FEI Company, operating at 15 kV) A comprehensive thermal analyzer (HCT-3, Beijing Hengjiu Scientific Instrument Factory) was employed to investigate the thermal properties of the samples, heating them from ambient temperature to 700°C at a heating rate of 10°C per minute.An infrared spectrometer (FT-IR, model 1.50 SU 1, Shimadzu Corporation), with a scanning range of 400–4000 cm^−1^, was utilized to acquire the infrared spectra of the samples.An X-ray diffractometer (XRD-7000, Shimadzu Corporation) was utilized to ascertain the crystal structure of the samples.An X-ray photoelectron spectrometer (XPS, Thermo ESCALAB 250XI, USA) was employed to ascertain the elemental composition and chemical valence states of the materials.Under conditions of an ambient temperature of 20°C, a degassing temperature of 100°C, and a degassing duration of 180 min, a specific surface area and pore size analyzer (3H-2000PS1, Builder Instrument Co.Ltd.) was utilized to measure the specific surface area and pore size distribution of the samples.The band gap of the samples was ascertained using solid-state ultraviolet diffuse reflectance spectroscopy (DRS).A HORIBA Fluorolog-QM transient fluorescence spectrometer (Canada) was employed to acquire the photoluminescence spectra (PL) of the samples at a wavelength of 425 nm and under room temperature conditions.An ultraviolet (UV) spectral analyzer was utilized to measure the laser intensity.*In situ* X-ray photoelectron spectroscopy (*in situ* XPS) was conducted using an ESCALAB 250 Xi (USA).Electrochemical impedance spectroscopy (EIS) was executed at an open-circuit voltage, with an amplitude of 10 mV and a frequency range from 10^−1^ to 10^5^ Hz for the assessment.

#### Analysis of adsorption performance

To evaluate the adsorption efficiency of the materials under different conditions, the initial concentration of tetracycline hydrochloride (TC) was set at 20 ppm. The effects of material type, temperature, and catalyst dosage on the adsorption performance were investigated respectively. The experiment was carried out in a dark environment to prevent light interference, and a magnetic stirrer was used to stir at a speed of 300 revolutions per minute to ensure uniform dispersion of the solution. During the 30-min reaction period, 2-mL samples were taken from the reactor at regular intervals and filtered through a membrane. An ultraviolet-visible spectrophotometer (UV-2250, Shimadzu Corporation, Japan) was used to measure the remaining TC content in the filtrate at a detection wavelength of 357 nm. The adsorption capacity of the materials was determined by evaluating their performance under different conditions and optimized parameters.

Based on the results of the adsorption test, 15 mg of the catalyst was selected for the photocatalytic degradation test. The catalyst was added to 30 mL of a tetracycline hydrochloride (TC) solution with a concentration of 20 mg/L. The resulting mixture was stirred in the dark for 30 min to achieve adsorption-desorption equilibrium. Subsequently, photocatalytic degradation experiments were carried out under different types and intensities of light (such as red laser, blue laser, mixed laser, ultraviolet light, and visible light). Samples were collected every 20 min. After filtering the samples to remove impurities, the concentration of TC was measured using an ultraviolet-visible spectrophotometer at a wavelength of 357 nm.

#### Catalytic degradation procedures

The degradation rate (DR) was calculated using the following formula: DR (%) = C_t_/C_0_× 100%, where C_0_ is the initial concentration after the dark reaction, and C_t_ is the concentration of the sample after degradation. The photocatalytic performance of the materials was evaluated by comparing their degradation rates under different lighting conditions. Figure 1 presents the laser-catalyzed degradation process of tetracycline hydrochloride.

Cycling stability is a key indicator for evaluating the practical application performance of photocatalytic materials. To evaluate the stability of the ZnO/Cu_1.35_O nanocomposite, four consecutive degradation cycles were carried out under the same experimental conditions. After each cycle, the material was separated by centrifugation, washed three times with methanol, and dried for the next degradation cycle.^53^

A radical trapping experiment was conducted to clarify the roles of different reactive species in the degradation of TC. L-histidine, p-benzoquinone, disodium ethylenediaminetetraacetate, and tert-butanol were used to scavenge singlet oxygen, superoxide anions, protons, and hydroxyl radicals, respectively. The photocatalytic degradation experiment was carried out under the following conditions: TC concentration of 20 mg/L, catalyst dosage of 0.5 g/L, and reaction time of 180 minutes.

#### Computational details

In all density functional theory (DFT) calculations for this study, the CASTEP module within Materials Studio software was employed for the optimization of geometric structures and the computation of electronic properties.The calculations utilized the Perdew-Burke-Ernzerhof (PBE) generalized gradient approximation (GGA) functional to treat the exchange-correlation potential, with a plane-wave cutoff energy set to 400 eV.The convergence criteria for geometric optimization were set as follows: residual forces between atoms less than 0.03 eV/Å, total energy change less than 1.0×10^-5^ eV, and atomic displacements less than 0.001 Å.To eliminate interactions between periodic image layers, a vacuum layer of 20 Å thickness was incorporated into the model.
